# Incorporation of tryptophan analogues into the lantibiotic nisin

**DOI:** 10.1007/s00726-016-2186-3

**Published:** 2016-02-12

**Authors:** Liang Zhou, Jinfeng Shao, Qian Li, Auke J. van Heel, Marcel P. de Vries, Jaap Broos, Oscar P. Kuipers

**Affiliations:** Department of Molecular Genetics, Groningen Biomolecular Sciences and Biotechnology Institute, University of Groningen, Nijenborgh 7, 9747 AG Groningen, The Netherlands; Laboratory of Biophysical Chemistry, Groningen Biomolecular Sciences and Biotechnology Institute, University of Groningen, Nijenborgh 7, 9747 AG Groningen, The Netherlands; Department of Pediatrics, University Medical Center Groningen, University of Groningen, Hanzeplein 1, 9713GZ Groningen, The Netherlands; Mass Spectrometry Core Facility, Department of Pharmacy, University of Groningen, Antonius Deusinglaan 1, 9713AV Groningen, The Netherlands

**Keywords:** Lantibiotics, Nisin, Tryptophan analogues, Biosynthetic incorporation, Non-canonical amino acids

## Abstract

Lantibiotics are posttranslationally modified peptides with efficient inhibitory activity against various Gram-positive bacteria. In addition to the original modifications, incorporation of non-canonical amino acids can render new properties and functions to lantibiotics. Nisin is the most studied lantibiotic and contains no tryptophan residues. In this study, a system was constructed to incorporate tryptophan analogues into nisin, which included the modification machinery (NisBTC) and the overexpression of tryptophanyl-tRNA synthetase (TrpRS). Tryptophan and three different tryptophan analogues (5-fluoroTrp (5FW), 5-hydroxyTrp (5HW) and 5-methylTrp (5MeW)) were successfully incorporated at four different positions of nisin (I1W, I4W, M17W and V32W). The incorporation efficiency of tryptophan analogues into mutants I1W, M17W and V32W was over 97 %, while the mutant I4W showed relatively low incorporation efficiency (69–93 %). The variants with 5FW showed relatively higher production yield, while 5MeW-containing variants showed the lowest yield. The dehydration efficiency of serines or threonines was affected by the tryptophan mutants of I4W and V32W. The affinity of the peptides for the cation-ion exchange and reverse phase chromatography columns was significantly reduced when 5HW was incorporated. The antimicrobial activity of IIW and its 5FW analogue both decreased two times compared to that of nisin, while that of its 5HW analogue decreased four times. The 5FW analogue of I4W also showed two times decreased activity than nisin. However, the mutant M17W and its 5HW analogue both showed 32 times reduced activity relative to that of nisin.

## Introduction

As the traditional antibiotics are not sufficient to deal with several drug-resistant pathogens (Ferri et al. 2015), many efforts have been made in mining new antimicrobial agents and engineering pre-existing antimicrobial peptides. Lantibiotics are gene-encoded and ribosomally synthesized peptides. After synthesis, they undergo various kinds of modifications conducted by enzymatic catalysis (Willey and van der Donk 2007). Lantibiotics are active mainly against Gram-positive bacteria (Bierbaum and Sahl 2009) and have been considered to possess pharmaceutical value in the future (Dischinger et al. 2014). The bioengineering of lantibiotics has been performed with various methods, including point mutation, modular shuttling, introducing new modifications and non-natural amino acids incorporation (Montalbán-López et al. 2012).

**Fig. 1:**
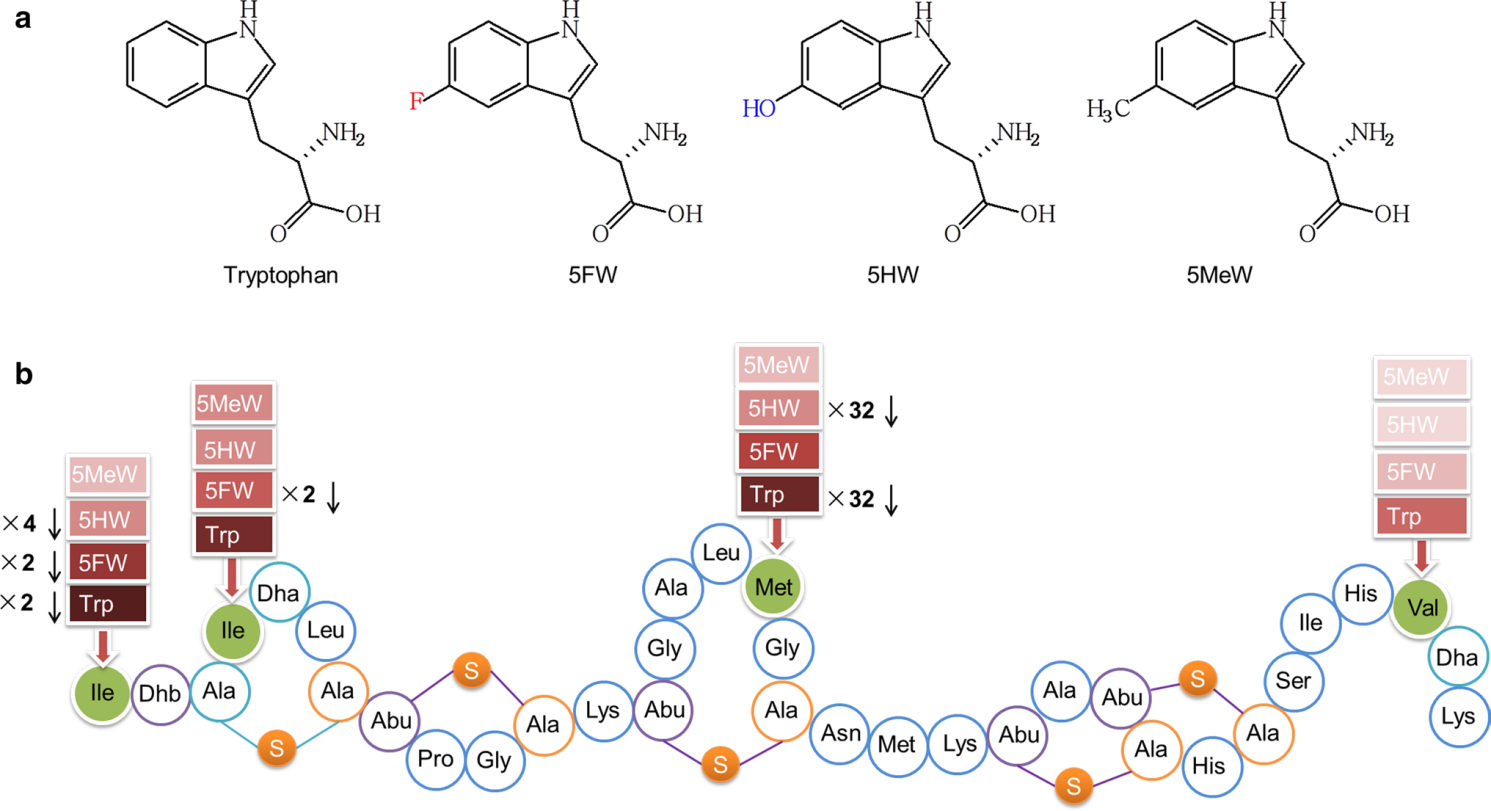
**a** Structures of tryptophan and its analogues used in this study. 5FW, 5-fluorotryptophan; 5HW, 5-hydroxytryptophan; 5MeW, 5-methyltryptophan. **b** Incorporation of tryptophan analogues into nisin A. Dha, dehydroalanine; Dhb, dehydrobutyrine; Ala-S-Ala, lanthionine; Abu-S-Ala, methyllanthionine. Ile1, Ile4, Met17 and Val32 (green label) were mutated and replaced by Trp or Trp analogues (*red label*). The colour depth of the *red label* indicates the yield of the variant and *darker colour* means higher yield. The antimicrobial activity of some variants compared to wild-type nisin is indicated. The yield of other variants was too low to purify enough material for accurate minimum inhibitory concentration (MIC) determination. In general, incorporation of a Trp analogue in nisin gave lower production yields and equal or higher MIC values than natural Trp incorporation

Nisin (Fig. 1b) is the first described lantibiotic and is produced by *Lactococcus lactis* (Rogers 1928). The biosynthesis of nisin (Lubelski et al. 2008) starts from a ribosomal synthesis of the prepeptide encoded by the *nisA* gene. The prepeptide consists of a leader part and a core peptide part. The leader part will guide the prepeptide to NisB which will catalyse the dehydration of serines or threonines in the core peptide part to dehydroalanines (Dha) or dehydrobutyrines (Dhb) by glutamyl-tRNA^Glu^-involved glutamylation and glutamate elimination (Ortega et al. 2015). The Dha or Dhb is then coupled to cysteine by a sulfhydryl addition reaction catalysed by NisC (Kuipers et al. 1993a; Koponen et al. 2002). NisB and NisC work interactively and directionally (Lubelski et al. 2009). The modified prenisin is transported outside the cell by an ABC transporter NisT (Qiao and Saris 1996; Kuipers et al. 2004).

Nisin can efficiently inhibit the growth of Gram-positive bacteria by two kinds of inhibition mechanisms. The first two rings of nisin can bind to lipid II which is essential for cell wall synthesis. The C-terminus of nisin can insert into the membrane enabling pore formation (Breukink and de Kruijff 2006). Mutagenesis of nisin has been conducted extensively and several residues have been mutated to tryptophan, e.g. I1W, M17W and V32W, which all reduce nisin activity but not dramatically, against *Micrococcus flavus* by two to three times and against *Streptococcus thermophilus* by three to eight times (Kuipers et al. 1995; Demel et al. 1996; Van Kraaij et al. 1997; Breukink et al. 1998).

In vivo incorporation of non-canonical amino acids (ncAAs) into proteins has been conducted in two ways (Hoesl and Budisa 2011). One is called “genetic code expansion” which means that non-canonical amino acids are inserted by suppressor tRNAs into the position where a termination codon exists. The other one is “genetic code engineering” which means canonical amino acids are replaced by their non-canonical analogues (i.e. ncAAs) during translation in an auxotrophic strain. Previously, Met, Trp and Pro analogues have been incorporated into a two-component lantibiotic lichenicidin using related auxotrophic *E. coli* strains (Oldach et al. 2012). Additionally, several kinds of lacticin 481 mutants containing ncAAs have been generated by in vitro mutasynthesis, i.e. the chemically synthesized substrate peptides containing ncAAs were modified by the enzyme LctM in vitro. Two of these lacticin 481 mutants containing Trp19Nal (Nal = naphthylalanine) or Phe23hPhe (hPhe = homophenylalanine) showed enhanced activity against *L. lactis* HP (Levengood et al. 2009).

Here, we investigate the in vivo incorporation of tryptophan analogues into nisin, as this could yield improved or altered-specificity variants. Previously, an efficient tryptophan analogue incorporation system for *Lactococcus lactis* has been reported (Petrović et al. 2013). In the *L. lactis* tryptophan auxotroph strain (PA1002), a native tryptophanyl-tRNA synthetase (lacTrpRS) was overexpressed. This TrpRS has a relaxed substrate specificity, which can increase the incorporation efficiency of Trp analogues and also improve the yield of the target protein. This system was combined with a system to incorporate lanthionine bridges based on the nisin modification machinery. We used one new (I4) and three previously published positions (1, 17 and 32) for the Trp codons. The latter ones have slightly higher MIC values than wild-type nisin.

In this research, three kinds of common tryptophan analogues (Fig. 1a) were successfully incorporated into nisin. The incorporation efficiency of Trp analogues was analysed with LC–MS. The new properties and antimicrobial activities of the variants are described.

## Materials and methods

### Bacterial strains and growth conditions

The bacterial strains used in this study are listed in Table 1. *L. lactis* strains were cultured in M17 broth supplemented with 0.5 % (w/v) glucose (GM17) or GM17 agar for genetic manipulation or in minimal expression medium (MEM) for protein expression at 30 °C (Rink et al. 2005a). Minimal chemical defined medium (mCDM) was specially used for protein expression and tryptophan analogue incorporation.

**Table 1:** Strains and plasmids used in this study

Strains or plasmids	Characteristics	Reference
Strain		
*Lactococcus lactis* NZ9000	*pepN::nisRK*	Kuipers et al. (1997)
*L. lactis* PA1001	Derivative of NZ9000, lacking *acmA* and *htrA*	Bosma et al. (2006)
*L. lactis* PA1002	Derivative of PA1001, lacking *trpB* and *trpA*	El Khattabi et al. (2008)
Plasmids		
pIL3EryBTC	*nisBTC*, encoding nisin modification machinery, EryR^a^	van Heel et al. (2013)
pCZ–nisA	*nisA,* encoding NisA, under the control of P*czcD* promoter, CmR^b^	Mu et al. (2013)
pMG36e–trpRS	*trpRS,* encoding tryptophanyl-tRNA synthetase (TrpRS), under the control of P32 promoter, EryR^a^	Petrović et al. (2013)
pCZnisA–trpRS	*nisA* and *trpRS*, encoding NisA and TrpRS, under the control of P*czcD* promoter, CmR^b^	This study
pCZnisA(I1W)–trpRS	Point mutant of pCZnisA–trpRS, with the Ile 1 of nisin changed to Trp	This study
pCZnisA(I4W)–trpRS	Point mutant of pCZnisA–trpRS, with the Ile 4 of nisin changed to Trp	This study
pCZnisA(M17W)–trpRS	Point mutant of pCZnisA–trpRS, with the Met 17 of nisin changed to Trp	This study
pCZnisA(V32W)–trpRS	Point mutant of pCZnisA–trpRS, with the Val 32 of nisin changed to Trp	This study
pNZnisP8H	*nisP*, encoding NisP mutant, with 8 histidines, CmR^b^	Unpublished data
Indicator strain		
*Lactococcus lactis* MG1363	Indicator strain	Gasson (1983)

^a^ *EryR* Erythromycin resistance

^b^ *CmR* Chloramphenicol resistance

### Genetic manipulation

The plasmid pCZnisA–trpRS was constructed by ligating the gene *trpRS* behind the *nisA* gene. The gene *trpRS* including the ribosomal binding site (RBS) and a terminator was cloned from the plasmid pMG36e–trpRS by performing round PCR (Zhou et al. 2015) with two primers containing BamHI and XhoI cleavage sites, respectively. The pCZnisA fragment was obtained by performing PCR with the reverse primer containing a BamHI cleavage site which binds to the sequence right behind the *nisA* gene. The forward primer was designed to amplify the full-length plasmid. The XhoI cleavage site is originally behind the terminator of *nisA* gene. Both PCR products were cut with BamHI and XhoI, and the digested products were purified (Roche Switzerland) and ligated with T4 ligase (Thermo Scientific). The ligation product was transformed into NZ9000 as described previously (Holo and Nes 1995). All the point mutants were made by round PCR using pCZnisA–trpRS as a template. For tryptophan analogue incorporation, the constructed plasmids pCZnisA–trpRS with or without mutants were transformed into PA1002-containing plasmid pIL3EryBTC.

### Incorporation of tryptophan analogues into nisin

The biosynthetic incorporation of Trp analogues is according to the paper of Petrović et al. (Petrović et al. 2013). The expression strain PA1002 harbouring the pIL3EryBTC plasmid and a pCZnisA–trpRS plasmid (Table 1) was cultured overnight in 5 ml M17 broth with 0.5 % (w/v) glucose, 5 µg/ml erythromycin and 5 µg/ml chloramphenicol at 30 °C. The overnight culture was then inoculated to the same medium (100 ml) and incubated at 30 °C. When OD600 reached 0.4–0.5, 2 nM nisin was added to induce the expression of NisBTC. After 3 h induction, cells were harvested by centrifugation at 5400*g* for 10 min. Subsequently, cells were washed with PBS three times and resuspended in 100 ml mCDM medium without tryptophan and with 3 µg/ml erythromycin, 3 µg/ml chloramphenicol and 0.2 nM nisin. After starvation at 30 °C for 30 min, 1 mM Trp analogues (5-methyl-tryptophan, 5-fluoro-tryptophan or 5-hydroxy-tryptophan) and 1 mM ZnSO_4_ were added. The culture was incubated at 30 °C overnight to express the nisin peptides. The supernatant was harvested by centrifugation at 9000 rpm during 10 min at room temperature or 4 °C. To remove the leader of the peptides before purification, the strain NZ9000 pNZnisPhis was fermented first with GM17 containing 5 µg/ml chloramphenicol until OD600 reached 0.7, and then the cells were harvested and resuspended in MEM with 0.5 % (w/v) glucose and 3 µg/ml chloramphenicol and induced with 2 nM nisin for 3 h. The supernatant was harvested by centrifugation (9000 rpm, 10 min).

### LC–MS of nisin variants with Trp or Trp analogues

4 ml supernatant was first concentrated by TCA precipitation (Link and LaBaer 2011) and then dissolved with 100 μl 0.01 % acetic acid. The concentrated peptides were analysed by FTMS using a Shimadzu UFLC system (Shimadzu, Den Bosch, The Netherlands) coupled online via the HESI interface with an LTC-Orbitrap-XL mass spectrometer (Thermo Fisher Scientific., San Jose, CA). 5 μl of the diluted samples was loaded onto a Shimpack XR-ODS2 column (2.0 × 50 mm, 2.2 μm particles). The following mobile phase gradient was delivered at a flow rate of 0.2 ml/min: 10 % B 1 min hold; linear gradient 10–35 % B in 19 min; 35–90 % B in 0.1 min; hold 90 % B 4.9 min. Solvent A was H_2_O (v/v) with 0.1 % formic acid and solvent B was 50:50 IPA/acetonitrile (v/v) with 0.1 % formic acid. Typical spray voltage was 3.5 kV [heater 250 °C, sheath gas flow 35 and auxiliary gas flow 10 (arbitrary units)]; ion transfer tube temperature was 300 °C. Analysis consisted of a high-resolution scan from *m/z* 350 to *m/z* 1800 with target mass resolution of 100,000 (FWHM, full width at half maximum at *m/z* 400). The obtained data were processed using the Thermo Xcalibur Xtract algorithm for deconvoluting isotopically resolved mass spectra.

## Purification and quantification of nisin variants with Trp or Trp analogues

To obtain pure peptides for activity tests, the supernatant of 100 ml culture (filtered with 0.2 µm filter, Millipore) was first incubated with 5 ml supernatant of NisP (filtered) at 30 °C for 1 h to cut off the leader part and then purified with cation-ion exchange (HiTrap SP FF, GE healthcare) (van Heel et al. 2013). The elutions were further loaded on a C18 column (Spherical C18, Sigma- Aldrich). The column was washed and eluted with different concentrations of solution B (solution A, milliQ with 0.1 % TFA; solution B, isopropanol: acetonitrile (2:1) with 0.1 % TFA). The elutions which were active against *L. lactis* were freeze-dried and further purified with HPLC as described previously (Zhou et al. 2015). The fractions containing the peaks in the HPLC chromatogram were analysed by activity tests and MALDI-TOF (van Heel et al. 2013). The peak which was active and contained the pure peptide with right mass was freeze-dried and quantified with HPLC as described previously (Zhou et al. 2015).

### Activity tests

The indicator strain *L. lactis* MG1363 was first streaked on a GM17 plate and cultured overnight. Before testing the MIC, three to five colonies were picked and cultured with GM17 medium until OD600 reached 0.5. The culture was diluted 500 times with GM17 and mixed with the same volume of peptides dissolved in GM17. The MIC value test was performed according to Wiegand et al. (2008).

## Results

### A new expression system to incorporate tryptophan analogues into nisin

**Fig. 2:**
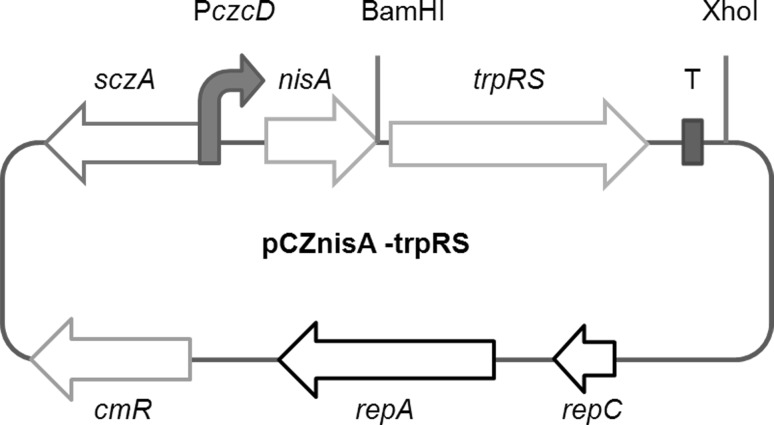
Map of the plasmid pCZnisA–trpRS. *sczA*, encoding the repressor of P*czcD*; P*czcD,* a zinc-inducible promoter; *nisA*, encoding NisA; *trpRS*, encoding tryptophanyl-tRNA synthetase; T, terminator; BamHI and XhoI, restriction sites; *repA* and *repC*, encoding plasmid replication proteins; *cmR*, chloramphenicol resistance gene. Partly referred to (Mu et al. 2013)

As nisin is a ribosomally synthesized and posttranslationally modified peptide, the expression and transportation of nisin need *nisABTC* gene expression. In the conventional nisin production system, both *nisA* and *nisBTC* gene were controlled by the P_*nis*_ promoter in separate plasmids pNZnisA and pIL3EryBTC (Rink et al. 2005b; van Heel et al. 2013). However, in this research, we wanted to control the expression of the modification enzymes separately from the expression of nisin, to limit the incorporation of tryptophan analogues into the modification machinery. As a second inducible promoter, we used zirex which is a tight and efficient zinc-inducible expression system for *L. lactis*. It contains a *pneumococcal* repressor SczA and P_*czcD*_ (Mu et al. 2013). In the new system, the P_*czcD*_ promoter was used to control the expression of nisin and its derivatives. In the system constructed by Petrović et al. (2013), the lacTrpRS protein was overexpressed and shown to be important for the incorporation. To incorporate tryptophan analogues into nisin more efficiently, lacTrpRS needs to be overexpressed. Previously, the *trpRS* gene is controlled by a constitutive promoter P32 in a separate plasmid pMG36e–trpRS. To avoid the need for three plasmids in one strain, the *trpRS* gene was cloned to plasmid pCZ–nisA, right behind the *nisA* gene and controlled by the P_*czcD*_ promoter (Fig. 2) as well. So in this new system, the expression of NisBTC is controlled by P_*nis*_ and could be conducted prior to the expression of NisA variants. The TrpRS and NisA variants were expressed at the same time, which can increase the incorporation rate.

### The variants were produced in different amounts, and are affected by the type of Trp analogues

**Fig. 3:**
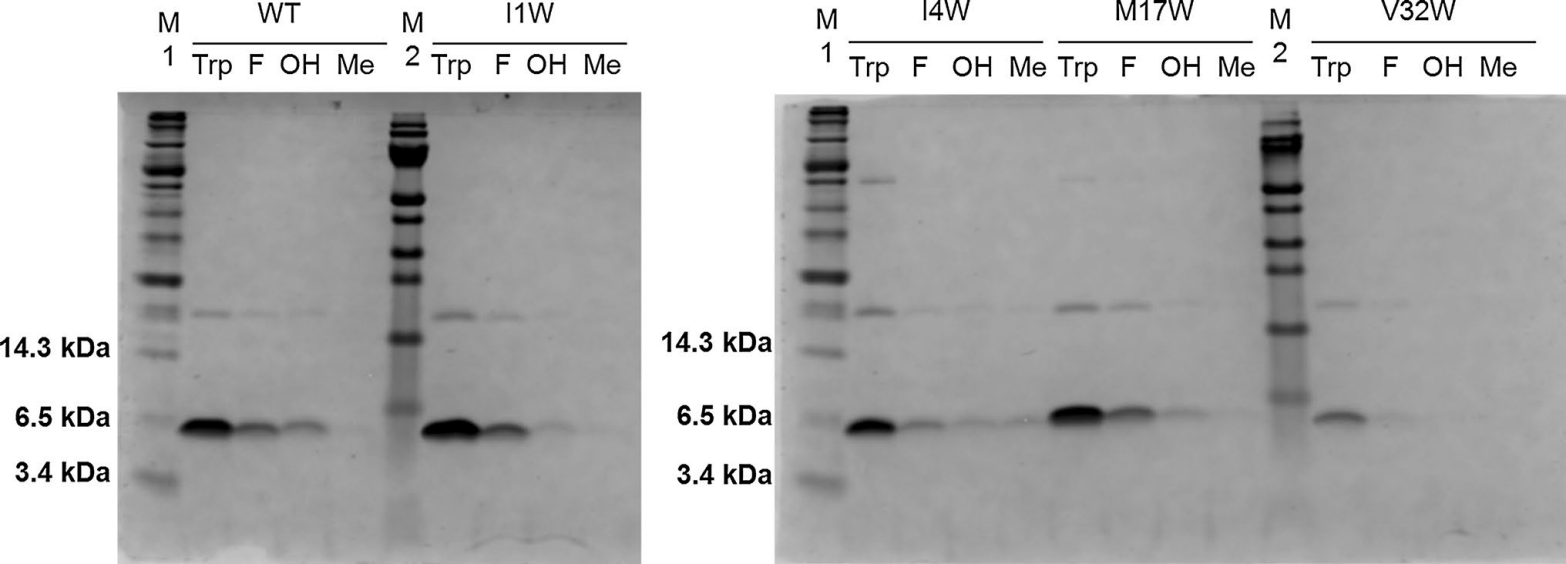
Coomassie blue-stained tricine SDS-PAGE gel. Each well contained TCA-precipitated prepeptides from 600 μl supernatant. M1: protein marker (Biolabs); WT: wild type; Trp: tryptophan; F: 5-fluoroTrp; OH: 5-hydroxyTrp; Me: 5-methylTrp

The nisin gene does not encode any tryptophan residue naturally. Therefore, nisin was engineered to encode tryptophan residues at four different positions (I1W, I4W, M17W and V32W (Fig. 1). These mutants were used to evaluate the incorporation efficiency of the tryptophan analogues at the different positions. With the new incorporation system, tryptophan and three different tryptophan analogues (5FW, 5HW or 5MeW) were incorporated into nisin. Figure 3 shows the expression level of wild-type nisin and four single-Trp-containing mutants of nisin cultured in the presence of Trp, 5FW, 5HW or 5MeW. The protein amount in the first four lanes showed that the wild-type nisin was expressed at a high level when tryptophan was in the medium, but in the presence of Trp analogues a lower production yield was observed. Especially when 5MeW was in the media, the production level was extremely low. The production level of all four nisin mutants showed almost the same trend as wild type, but mutant V32W showed much lower production level than wild type and the three other mutants.

### The variants were correctly modified and the Trp analogues were incorporated

As the TCA-precipitated prepeptides from supernatant contain various kinds of peptides secreted by the cells, they were used as samples for LC–MS analysis. The first Met is normally removed in the mature product of prenisin (Kuipers et al. 1993b) by a methionine aminopeptidase (MAP) (Sherman et al. 1985). However, a large portion of prepeptides produced by this system contained an N-terminal Met. As the peptides with or without Met1 showed almost the same ratio between peaks of different modifications, only the peaks without Met1 are shown in Table 2.

**Table 2:** The molecular mass of the nisin mutants after tryptophan analogues are incorporated

Mutants	Modifications	Predicted mass (Da)	Observed mass (Da) of main peaks at different culture conditions			
			Trp	5FW	5HW	5MeW
I1W	−8H_2_O	5757.750	5757.747			
	+F −8H_2_O	5775.741		5775.752		
	+OH −8H_2_O	5773.745			5773.737	
	+Me −8H_2_O	5771.766				5771.752
I4W	−8H_2_O	5757.750	5757.735 (7)^a^	5757.776 (9)	5757.795 (2)	5757.789 (30)
	−7H_2_O	5775.761	5775.724 (69)			
	−7H_2_O + Oxi	5791.756	5791.726 (24)			
	+F −8H_2_O	5775.741		5775.734 (59)		
	+F −8H_2_O + Oxi	5791.736		5791.733 (30)		
	+OH −8H_2_O	5773.745			5773.766 (35)	
	+OH −7H_2_O	5791.756			5791.753 (14)	
	+OH −8H_2_O + Oxi	5789.740			5789.751 (24)	
	+OH −8H_2_O + 2Oxi	5807.751			5807.743 (20)	
	+Me −8H_2_O	5771.766				5771.758 (33)
	+Me −7H_2_O	5789.776				5789.765 (36)
M17W	−8H_2_O	5739.794	5739.763			
	+F −8H_2_O	5757.784		5757.791		
	+OH −8H_2_O	5755.794			5755.727 (30)	
	+OH −8H_2_O + Oxi	5771.789			5771.751 (65)	
	+Me −8H_2_O	5753.809				5753.749
V32W	−8H_2_O	5771.766	5771.757 (30)			
	−7H_2_O	5789.776	5789.768 (60)			
	+F −8H_2_O	5789.756		5789.752 (14)		
	+F −7H_2_O	5807.767		5807.759 (36)		
	+F −8H2O + Oxi	5805.751		5805.754 (8)		
	+F −7H2O + Oxi	5823.762		5823.756 (33)		
	+OH −8H_2_O	5787.761			ND^b^	
	+Me −8H_2_O	5785.781				ND^b^

^a^ The values between brackets refer to the percentage of different variants; similarly hereinafter

^b^ *ND* Not detected

The incorporation efficiency indicates the ratio between the amount of peptides with tryptophan analogues and the total amount of peptides. The LC–MS data show that the incorporation efficiency of tryptophan analogues into mutants I1W, M17W and V32W was more than 97 %, as the peaks of peptides containing Trp were almost undetectable. But in the case of I4W, a significant Trp variant peak was shown, and the incorporation efficiency was 89 % for 5FW, 93 % for 5HW and 69 % for 5MeW. Nisin is normally dehydrated eight times, but seven or six times dehydration could also be observed. More than 70 % of the mutants I1W and M17W were dehydrated eight times, which is similar to the dehydration extent of nisin. However, the dehydration of I4W and V32W was dramatically affected by the mutation of Trp. 93 % of the I4-W variant was dehydrated seven times, while when Trp analogues were incorporated the eight times dehydrated peptides occupied a larger proportion. In the case of V32W, 60 % (Trp) or 69 % (5FW) of the products were dehydrated seven times. Furthermore, as there are two Met residues in nisin, oxidation of Met happens in a fraction of the product. About 24–44 % of the products of I4W were oxidized. I4-5HW was oxidized two times in 20 % of the product. In the case of M17W incorporated with 5HW, about 65 % of the product was oxidized. When 5FW was incorporated into the mutant V32W, about 40 % of the peptides were oxidized. V32-5HW and V32-5MeW were not analysed because of the low production level.

### The tryptophan analogues change the properties of nisin

Nisin is a positively charged and hydrophobic molecule. However, when introducing Trp or Trp analogues to it, nisin showed modified biochemical properties. This difference was reflected by the elution time of the peptides in cation-ion exchange and reverse phase chromatography (HPLC). The variants with 5HW were eluted about half column volume earlier than other variants during cation-ion exchange (data not shown). The variants with Trp, 5FW or 5MeW showed slightly different elution time from nisin in HPLC. But the 5HW-containing variants showed much shorter retention time than other variants (Table 3).

**Table 3:** Retention time of the nisin variants in HPLC

Nisin variants	Retention time of HPLC (min)
Nisin	38.0
I1-W	38.0
I1-5FW	38.9
I1-5HW	35.0
I1-5MeW	38.0
I4-W	38.4
I4-5FW	37.2
I4-5HW	34.8
M17-W	38.5
M17-5FW	39.1
M17-5HW	37.2
V32-5FW	39.6

### Antimicrobial activity of the variants containing tryptophan analogues

**Fig. 4:**
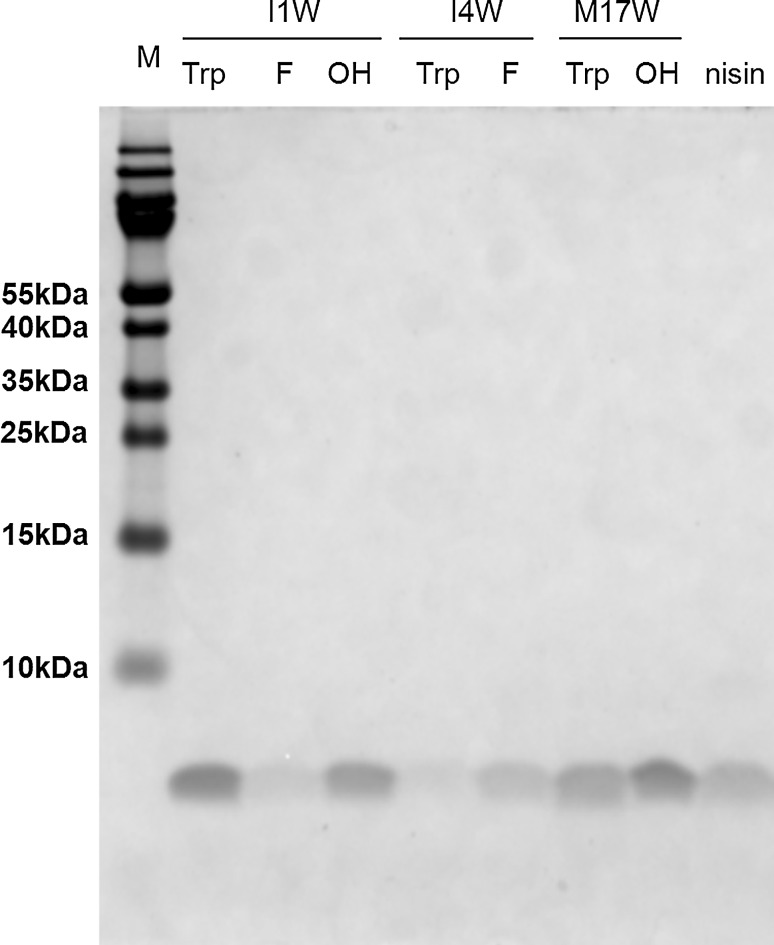
Purified tryptophan analogues-containing variants shown by Coomassie blue-stained tricine SDS-PAGE gel

Because of shortage of material, part of the tryptophan analogues containing variants were purified (Fig. 4) and their antimicrobial activity was tested against *L. lactis* (Table 4). The results showed that the mutant I1W displayed only two times reduced activity than nisin, and incorporating 5FW analogue into this mutant did not change the activity. But the 5HW analogue of I1W displayed two times lower activity than I1W. As fully dehydrated I4W variant was difficult to obtain, the activity of I4W was not tested. The mutant I4W with 5FW incorporated contained 30 % oxidized variants and the peptides showed two times reduced activity compared to nisin. However, the M17W mutant and its 5HW incorporated variants displayed dramatically decreased (32 times) activity.

**Table 4:** Antimicrobial activity of the tryptophan analogues-containing variants

Nisin variants	Predicted mass (Da)	Observed mass(Da)	Modifications	MIC against *L. lactis* MG1363 (nM)^a^
nisin	3352.5	3352.5	−8H_2_O	64
I1-W	3425.5	3425.6	−8H_2_O	128
I1-5FW	3443.5	3443.5	−8H_2_O	128
I1-5HW	3441.5	3441.5	−8H_2_O	256
I4-5FW	3443.5	3443.5	−8H_2_O (70 %)	128
	3459.5	3459.5	−8H_2_O + Oxi (30 %)	
M17-W	3407.6	3407.6	−8H_2_O	2048
M17-5HW	3423.6	3423.6	−8H_2_O	2048

^a^The MIC tests were repeated three times

## Discussion

Incorporation of non-canonical amino acids into natural products can be a strategy to improve the diverse properties of these compounds. For this purpose, an in vivo nisin—tryptophan analogue incorporation system in *L. lactis* was created. Three different tryptophan analogues have been successfully incorporated at four different positions of nisin. In contrast to the in vitro incorporation (Levengood et al. 2009) and the in vivo incorporation system in *E. coli* (Hoesl and Budisa 2011), this is the first system constructed in *L. lactis* to incorporate unnatural amino acids into lantibiotics in vivo.

**Fig. 5:**
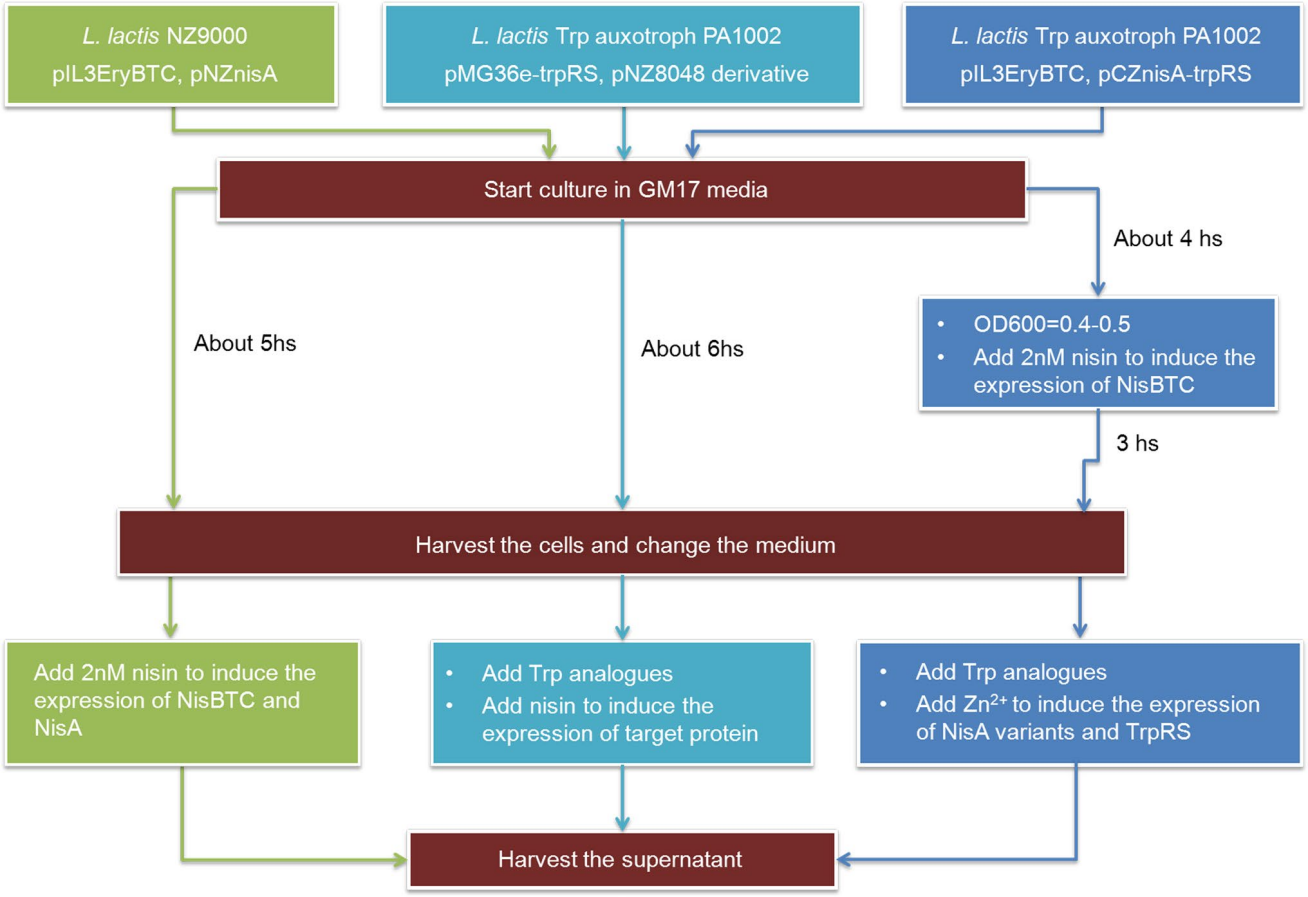
Nisin variant production and tryptophan analogue incorporation systems. *Olive line* the conventional NICE system; *aqua line* the tryptophan analogues incorporation system constructed by Petrović et al.; *blue line* the new system constructed in this research, which combines the NICE system, zirex system and the tryptophan analogues incorporation system

The newly constructed lanthionine and tryptophan analogue incorporation system has several advantages. The combination of the zirex and the nisin-controlled gene expression system (NICE) allows for differential timing of the expression of the lantibiotic and its modification enzymes. Figure 5 shows three different nisin variants production and tryptophan analogue incorporation systems. The enzymes NisBTC contain 17 Trp residues, and in the system presented in this work they were expressed in advance, which can avoid the Trp analogues being incorporated into NisBTC. This can thus accumulate large amount of modification enzymes before the expression of prenisin variants. The TrpRS, a tryptophan-less protein, was overexpressed together with prenisin, which is different from the original system for tryptophan analogue incorporation where the TrpRS was controlled by a constitutive promoter P32.

Both wild-type nisin and the mutants showed good production levels when tryptophan was used to culture the cells. This indicates that the new system works efficiently in the production and modification of nisin variants. However, the expression levels were reduced dramatically when the tryptophan analogues were in the medium. This is probably because the tryptophan analogues are toxic to the cells. If the tryptophan analogues were incorporated into NisBTC, the function of NisBTC could be affected and the production level could be reduced.

The incorporation efficiency of three kinds of Trp analogues into mutants I1W, M17W and V32W was over 97 %. This is similar to that reported previously (Petrović et al. 2013), which showed an incorporation efficiency of 98 % for 5FW, and 95 % for 5HW and 5MeW. These data indicate that the incorporation system and the incorporation method are efficient and widely applicable.

Although NisBTC was expressed in advance, the accumulated NisBTC performed the modification and transportation correctly. The mutants I4W and V32W variants showed a one time less dehydration extent, which is probably because the serines besides the tryptophan residues escaped dehydration. All mutants were cut well by NisP, which indicates that the mutation (especially I1W) and the analogues did not affect the activity of NisP.

Incorporating non-canonical amino acids into peptides is a method to introduce new properties into the peptides. The 5HW incorporation significantly changed the column desorption properties of nisin, which is probably because of the additional hydrogen bonding properties and an increased polarity of this analogue compared to Trp.

The first two rings of nisin can bind to lipid II by forming a pyrophosphate cage (Hsu et al. 2004). Against *L. lactis*, the I1W mutant showed only a twofold reduced activity, which is similar to the activity tested previously against *M. flavus* (Kuipers et al. 1995; Demel et al. 1996; Breukink et al. 1998). This indicates that the first Ile is not crucial for the binding. 5FW labelled I1W showed the same activity, which means that the fluorine group did not change the property of the peptide significantly. 5HW labelled mutant I1W showed two times lower activity than I1W, which means that the hydroxyl is detrimental for the binding activity of nisin. Ile4 plays an important role in the pyrophosphate cage formation (Hsu et al. 2004). However, 5FW labelled mutant I4W only modestly reduced the activity, which is probably because a pyrophosphate cage is formed by the backbone amides of Ile4 and the side chain of residue 4 does not significantly affect the binding. The MIC of I4W was not tested, but from the case of I1W and I1-5FW, the fluorine group would not change the activity significantly. Mutation of Met17 into Trp or 5HW decreased the activity dramatically (32 times). This suggests that the new bulkier side chain of residue 17 is too bulky to go into the membrane; therefore the binding efficiency and the pore formation activity of the peptide was reduced. In the previous study, the M17W mutant of nisin Z displayed only two times reduced activity against *M. flavus* and eight times reduced activity against *S. thermophilus* (Demel et al. 1996; Breukink et al. 1998), and the difference in activity might be related to the different indicator strains.

Nisin cannot be detected by UV at 280 nm because it contains no tryptophan or tyrosine residues. Introducing tryptophan or tryptophan analogues into nisin has the advantage of the UV detection being used during purification and for quantification. Furthermore, there are various kinds of tryptophan analogues, which show different properties and functions. If they could be incorporated into nisin, the activity and other characters of nisin might be improved.

Taken together, this research has opened the door to efficiently incorporate tryptophan analogues into nisin. The expression platform used here, Trp auxotroph PA1002 coexpressing lacTrpRS, is the most versatile Trp analogue incorporation system known, able to incorporate a large variety of Trp analogues as well as an amino acid containing the azulene moiety. Most of these analogues are commercially available or can be readily synthesized. In future studies, more residue positions should be targeted and the antimicrobial activity of the generated nisin peptides investigated. These structure function studies will also provide valuable insight into the mechanism of action of nisin.
